# Left Hemifacial Spasms Due to Left Vertebrobasilar Dolichoectasia

**DOI:** 10.7759/cureus.60081

**Published:** 2024-05-11

**Authors:** Sanjay M Khaladkar, Prajakta P KirdatPatil, Aryaman Dhande, Neeha A Jhala, Suhas M

**Affiliations:** 1 Radiodiagnosis, Dr. D. Y. Patil Medical College, Hospital & Research Centre, Dr. D. Y. Patil Vidyapeeth, Pune, IND

**Keywords:** botulinum toxin injection, cerebellopontine cistern, facial nerve involvement, vertebrobasilar dolichoectasia, hemifacial spasms

## Abstract

Hemifacial spasm (HFS) arises from involuntary, recurrent, irregular tonic-clonic-like contractions of the muscles innervated by the facial nerve. Typically, compression of the facial nerve root exit on the same side is attributed to either a vascular loop or a mass located in the cerebellopontine (CP) angle. Dolichoectasia, alternatively termed dilated arteriopathy, is characterized by arterial dilatation, elongation, and tortuosity. Here, we present a case involving vertebrobasilar dolichoectasia (VBD) as the cause of HFS, alongside relevant imaging findings.

## Introduction

Hemifacial spasm (HFS) is characterized by involuntary, repetitive, and irregular contractions of the facial muscles innervated by the facial nerve, resulting in involuntary and painless spasms. It usually starts with spasms affecting the orbicularis oris muscle and gradually extends to affect other facial muscles. Its occurrence in the United States varies from 0.5 to 2.4 per 100,000 people [1]. Tortuous blood vessels, specifically the posterior inferior cerebellar artery (PICA) and anterior inferior cerebellar artery (AICA), are the primary culprits for the compression of the facial nerve [2]. Vertebrobasilar dolichoectasia (VBD) causing direct compression is a rare cause of facial nerve compression. The usual complications of VBD include ischemic stroke (17.6%), brainstem compression (10.3%), and transient ischemic attack (10%) [3]. Cerebral microbleeds are also commonly observed in patients with VBD.

## Case presentation

A 70-year-old female presented with a left HFS persisting for the past six months. She reported no upper or lower limb weakness and no other neurological deficits. No other relevant complaints were disclosed by the patient. The patient was a known hypertensive for the past 20 years.

**Figure 1:**
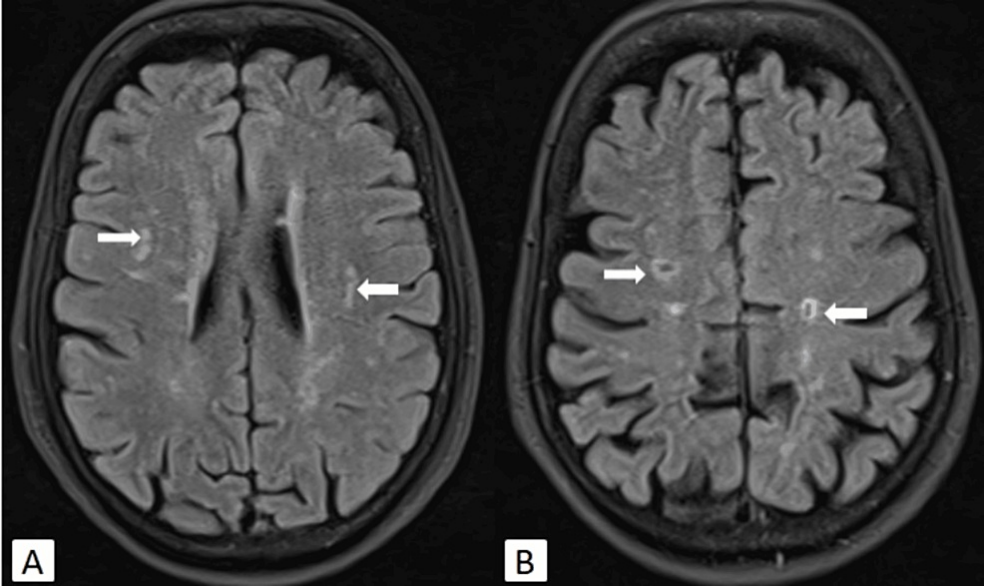
MRI of the brain axial FLAIR images showing white matter ischemic changes (A) and old lacunar infarcts (B) in the bilateral periventricular white matter and centrum semiovale region (white arrows). FLAIR: fluid-attenuated inversion recovery; MRI: magnetic resonance imaging

Magnetic resonance imaging (MRI) of the brain revealed multiple hyperintense foci in the fronto-parietal periventricular white matter and centrum semiovale region on long repetition time (TR) images and fluid-attenuated inversion recovery (FLAIR). These foci did not show diffusion restriction on diffusion-weighted imaging (DWI), indicative of chronic ischemic changes (Fazekas score 1). Old lacunar infarcts were noted in the bilateral corona radiata and centrum semiovale regions (Figure 1). No acute infarct or intracerebral hemorrhage was observed on DWI and susceptibility-weighted imaging (SWI) sequences, respectively.

**Figure 2:**
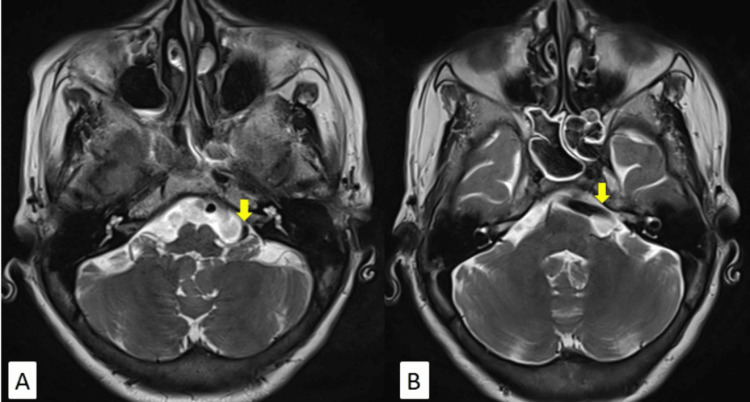
(A, B) MRI of the brain axial T2WI showing dolichoectasia of the left vertebral artery (yellow arrows) compressing the left VII-VIII nerve complex in the left CP angle cistern. T2WI: T2-weighted imaging; CP: cerebellopontine; MRI: magnetic resonance imaging

Axial T2-weighted imaging (T2WI) showed an ectatic left vertebral artery encroaching left cerebellopontine (CP) angle cistern causing mild compression over the left VII-VIII nerve complex (Figure 2).

**Figure 3:**
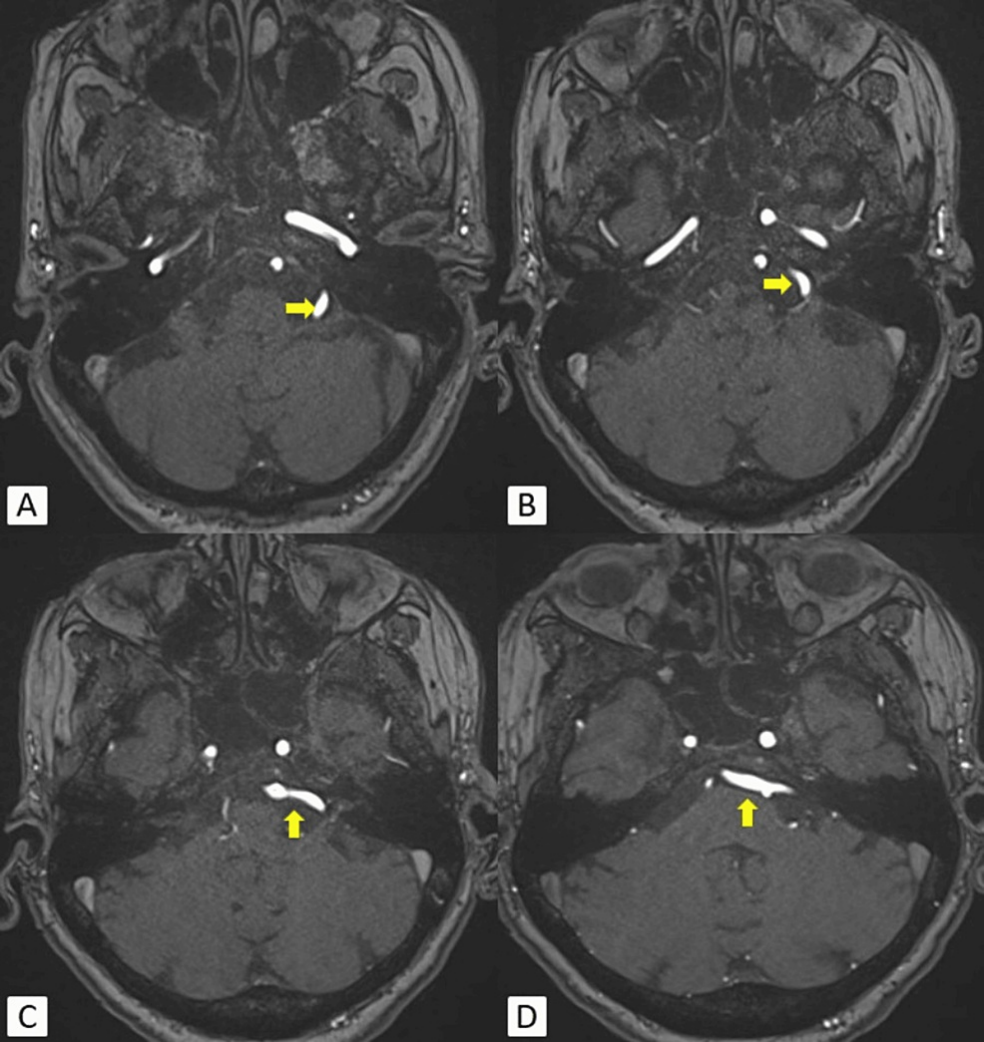
(A-D) 3D TOF MRI of the brain angiography showing dolichoectasia of the left vertebral artery (yellow arrows) extending in the left CP angle cistern causing mild compression over the left VII-VIII nerve complex. TOF: time of flight; CP: cerebellopontine; MRI: magnetic resonance imaging

Magnetic resonance angiography (MRA) of the brain using 3D time of flight (TOF) revealed dolichoectasia of the left vertebral artery. It was encroaching and causing mild compression of the cisternal segment of the left VII-VIII nerve complex (Figure 3). Type 2 left AICA was also noted. The rest of the study showed normal findings.

**Figure 4:**
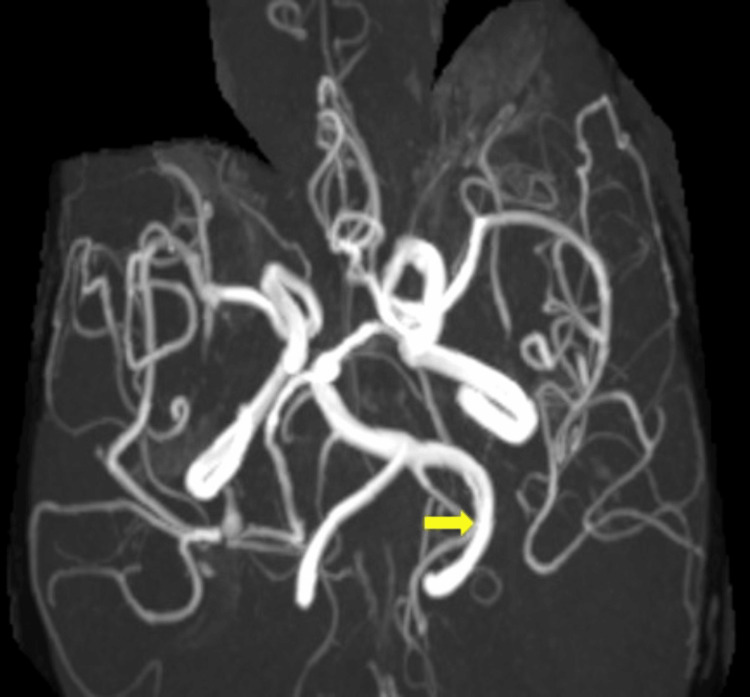
MRA of the brain MIP image showing dolichoectasia of the left vertebral artery (yellow arrow) extending in the left CP angle cistern. MIP: maximum intensity projection; MRA: magnetic resonance angiography; CP: cerebellopontine

MRA of the brain maximum intensity projection (MIP) image shows dolichoectasia of the left vertebral artery extending in the left CP angle cistern (Figure 4).

Using an insulin syringe, the patient received an injection of botulinum toxin (50 units in 1.1 ml of normal saline), which provided symptomatic relief for three months. However, during the follow-up after three months, the patient started experiencing similar symptoms and opted for Ayurvedic treatment instead. 

## Discussion

VBD is an intra-arterial condition characterized by the elongation and dilatation of the vertebral and basilar arteries. It can be acquired or hereditary. Risk factors for acquired intra-arterial dolichoectasia include aging, hypertension, and male gender. VBD can lead to various symptoms, such as HFS, tinnitus, and brainstem compression [4]. HFS, although rare, can be a cause of movement disorder and may induce psychological stress [5]. Therefore, accurate diagnosis and appropriate management are crucial [6]. Worldwide, its prevalence is reported to be 14.5 per 1 lakh in women and 7.4 per 1 lakh in men. Compression of the facial nerve can be attributed to tortuous arteries such as the AICA, PICA, and superior cerebellar artery (SCA) [6-8]. Direct compression by VBD is rare, with only 0.7% of cases reported in a study involving 1642 cases of HFS [9,10]. Advances in imaging techniques allow for the diagnosis of this condition by visualizing the compression of the facial nerve at its root entry zone as it emerges from the brainstem. MRI and MRA with thin T2 constructive interference in steady-state (CISS) sequences are highly effective in diagnosing this condition. When the root entry zone is compressed, it generates antidromic impulses, which lead to HFS. The result is an excitation of the face nucleus, known as a kindling phenomenon [11]. HFS caused by vascular compression by VBD presents two treatment options: botulinum toxin injection, known for its low incidence of adverse effects, which prolongs the duration of treatment for up to 20 years, and microvascular decompression. Microvascular decompression offers a definitive treatment but carries complications such as facial palsy and hearing impairment. It serves as a viable and effective option for patients previously treated with botulinum toxin. Botulinum toxin injections around the eye and cheek provide symptomatic relief with minimal side effects. However, the disadvantage lies in the need for repeated injections every few months indefinitely. On the other hand, surgical microvascular decompression offers the advantage of a permanent solution but comes with serious complications, including deafness (2.7%), facial paralysis (1.7%), cerebellar hematoma (0.5%), brainstem infarct (0.3%), and death (0.1%) [12].

## Conclusions

HFS commonly arises from facial nerve root exit compression, frequently attributed to vascular loops or masses situated in the CP angle. VBD is characterized by the elongation and dilatation of the vertebral and basilar arteries, either acquired or hereditary. It can lead to various symptoms, including HFS, tinnitus, and brainstem compression. The prevalence of HFS caused by VBD is rare, but accurate diagnosis is crucial for appropriate management. Advances in imaging techniques, such as MRI and MRA with thin T2 CISS sequences, facilitate the diagnosis of VBD and its compression of the facial nerve.
